# Organizational Readiness for Community Health Worker Workforce Integration Among Medicaid Contracted Health Plans and Provider Networks: An Arizona Case Study

**DOI:** 10.3389/fpubh.2021.601908

**Published:** 2021-06-07

**Authors:** Samantha Sabo, Nancy Wexler, Louisa O'Meara, Heather Dreifuss, Yanitza Soto, Floribella Redondo, Heather Carter, Jill Guernsey de Zapien, Maia Ingram

**Affiliations:** ^1^Center for Health Equity Research, Northern Arizona University, Flagstaff, AZ, United States; ^2^The John A. Hartford Foundation, New York, NY, United States; ^3^Department of Health Promotion Sciences, Zuckerman College of Public Health, Tucson, AZ, United States; ^4^Arizona Department of Health and Human Services, Phoenix, AZ, United States; ^5^Arizona Community Health Worker Association, Douglas, AZ, United States

**Keywords:** Community Health Workers, integration, health systems, recruitment, retention

## Abstract

Understanding and building organizational capacity for system change and the integration of the Community Health Worker (CHW) workforce within the health scare sector requires a supportive organizational culture among sector leaders and providers. The aim of this mixed-methods study was to assess organizational readiness for CHW workforce integration into Arizona Medicaid health systems and care teams. This collaborative effort was in direct response to emergent state and national CHW workforce policy opportunities, and the shifting health care landscape in Arizona – which merged behavior and physical health. Specifically, and in collaboration with a broad-based, statewide CHW workforce coalition, led by the CHW professional association, we assessed 245 licensed health care professionals with experience working with CHWs and 16 Medicaid-contracted health plan leadership. Our goal was to generate a baseline understanding of the knowledge, attitudes and beliefs these stakeholders held about the integration of CHWs into systems and teams. Our findings demonstrate a high level of organizational readiness and action toward integration of CHWs within the Arizona health care system and care teams. CHWs have emerged as a health care workforce able to enhance the patient experience of care, improve population health, reduce cost of care, and improve the experience of providing care among clinicians and staff.

## Introduction

The national expansion of health plans and health-plan contracted provider groups that promote the use of the Community Health Worker (CHW) workforce within clinical care has increased in the last decade. More markedly with the proliferation of the Quadruple Aim framework, which acknowledges the critical role of the health care team in healthcare transformation (1), CHWs have emerged as a health care workforce able to enhance the patient experience of care, improve population health, reduce cost of care, and improve the experience of providing care among clinicians and staff (2, 3). The inclusion of CHWs in multidisciplinary care teams contributes to the efficacy of Patient-Centered Medical Homes (PCMH), Accountable Care Organizations (ACO) and Community Health Teams (4–6). In addition to coordinated care, both ACOs and PCMHs are required to provide routine preventive care and patient education. CHWs are documented to be well-positioned to support these entities and effectively meet health reform mandates for prevention, education and coordination of care (4, 5). Movement toward Medicaid financing for value-based purchasing, or health plan reimbursement for patient population outcomes rather than per capita health services, offers yet another opportunity for the integration of CHWs into health systems and as members of the care team.

Medicaid health plans have also begun to act on opportunities presented by population-driven, value-based provider contracting to expand and promote CHW activities. Several state Medicaid programs, including Alaska, Minnesota (7) and Oregon, have specifically named CHWs as core participants in health care delivery reform. Oregon's Medicaid administered Coordinated Care Organization (CCO) payer-provider partnerships require the integration of CHWs in the healthcare team and train several 100 CHWs to support its CCOs (8). These actions come after the monumental 2014 decision by the Centers for Medicaid and Medicare (CMS) to issue guidance to allow states to reimburse for preventive services offered by non-licensed professionals such as CHWs (9). Yet, few states have taken advantage of policy opportunities to establish permanent financing systems to integrate CHWs formally into the health care delivery system.

Understanding and building organizational capacity for systems change and integration of CHWs within the health care sector requires attention to the organizational culture of the health care sector and the actors operating within in it (10). Organizational culture is most often defined by the collective behaviors, values, beliefs, attitudes and norms of the system and its actors (10). Often, leadership is at the core of organizational cultural. Here, in response to this special research topic on integration of CHWs within systems and teams, we aim to address the topic of organizational readiness to ensure successful integration of CHWs into health care systems and teams.

### Arizona Context

Arizona's Medicaid health care delivery system has a growing interest in the potential for the CHW workforce to impact health outcomes and costs, motivated at least in part by considerable policy shifts in the delivery of health care. In October 2018, Arizona's Medicaid system, known as Arizona Health Care Cost Containment System or AHCCCS, implemented the Arizona Complete Care (ACC). ACC requires Medicaid-contracted health plans to integrate behavioral and physical health services within one delivery mode, affecting ~1.5 million or ~80% of Medicaid members in Arizona (11). In the same year, Arizona's CHW workforce gained a substantial policy win through the passage of HB2324, providing the pathway and infrastructure for CHW voluntary certification and mandating CHW-driven workforce standards in training, supervision and career progression (12). While the current policy environment in Arizona is conducive to the integration of CHWs into health systems and clinical care teams, individual and systems-level barriers may hinder the potential for CHW integration to positively impact health outcomes (13–15).

In direct response to emergent state and national CHW workforce policy opportunities, and the shifting health care landscape in Arizona, we aimed to engage the Medicaid-focused health care sector of Arizona. Specifically, we assessed health care sector actors critical to organizational readiness and systems change: licensed health care professionals with experience working with CHWs and Medicaid-contracted health plan leadership. Our goal was to generate a baseline understanding of the knowledge, attitudes and beliefs these stakeholders held about the integration of CHWs into systems and teams.

## Materials and Methods

This study was guided by members of the Arizona Community Health Worker Coalition inclusive of more than 100 CHW stakeholders, including the Arizona Community Health Worker Association, CHW employers, the Arizona Department Health Services, Universities and health policy experts among many others. As a partnership, we have been engaged in several CHW workforce assessments with a focus on systems and environmental change that benefit the workforce as a whole. Between 2015 and 2019, we implemented a mixed-methods study with multiple aims: assessing organizational readiness, onboarding processes and integration of CHWs into Arizona Medicaid health systems and teams, as well as the perceived impact of CHW integration on health outcomes and cost of care among licensed providers in the state. This collaborative study took place during a period of a fast-moving CHW workforce policy landscape in the state.

### Health Provider Survey

In 2015, to assess organizational readiness for system change and actual integration of CHWs, we implemented a cross-sectional, on-line survey with Arizona licensed health providers Table 1. Our aim was to engage Arizona licensed providers to assess their knowledge, beliefs, attitudes and professional experience with the CHW workforce, the perceived influence of CHWs on patient outcomes and the quality and cost of care, and the mechanisms for CHW integration within the health care team. Our brief survey was adapted from and developed in collaboration with national CHW workforce policy experts with experience in surveying health care providers and systems leaders in Massachusetts, Texas and Wisconsin. Our survey was piloted with local, busy primary care providers employed in Federally Qualified Community Health Centers (FQHC) and adjusted to take no more than 5 min. We disseminated the survey by email and face-to-face to the universe of licensed providers in Arizona serving the Medicaid population and or employed within health systems that commonly employ CHWs in Arizona. This universe of providers included all 22 FQHCs, the three Indian Health Service (IHS) Areas of Arizona and all tribal health centers and clinics, as well as various behavioral health centers, local and state health provider professional associations and networks (i.e. family medicine, nursing, social work, pharmacists). Survey questions are detailed in Table 1 and followed a 5-point Likert scale ranging from strongly agree to strongly disagree. Variables were collapsed into three categories: Agree, Unsure and Disagree. Due to very low percentages within the Disagree category, data is presented as Agree and the remaining proportions are Unsure. Analysis was performed using Stata Statistical Software. Descriptive statistics were generated to characterize patient demographics and stratify by provider practice type, including (1) Federally Qualified Community Health Center, (2) Indian Health Service/638 Clinic/Hospital, (3) Health Practice and (4) Other Health Practice (inclusive providers employed in solo practice, group practice, managed care organization, and or hospital-based practice.

**Table 1:** Health plan interview domain and licensed provider survey questions.

**Instrument**	**Topic areas**
**Health plan leadership interview guide (*****N*** **= 16)** Semi-structured, qualitative small group phone interviews with 2-3 members of the health plan leadership team.	I. Familiarity and involvement with CHWs II. Utilization of CHWs in the Health Plan and contracted provider network 1. Roles for CHWs 2. Motivation for using CHWs 3. Qualifications/Identification/Recruitment 4. Training CHWs 5. Length of time using CHWs 6. Challenges in hiring and/or integrating CHWs into Health Plan workforce III. Determining the value of CHWs in care management 1. Importance of CHWs in improving *quality* of care 2. Importance of CHWs in improving *cost* of care 3. Most valuable contribution of CHWs to health plan/networks 4. Evaluation of cost savings or quality of care improvement 5. Timeframe for demonstrating impact of CHWs 6. CHW influence in designation of High Value/Center of Excellence IV. Payment models to support CHWs 1. Financing/How health plans pay to use CHWs as part of health care team 2. How provider networks are using CHWs to achieve value-based incentives 3. Interest in and financing of CHWs in community-based positions V. Current Arizona law/policies relating to CHWs 1. Value of CHW Voluntary Certification (HB2324) to health plans 2. Impact of AHCCCS Complete Care (ACC) on use of CHWs
**Licensed health provider survey (*****N*** **= 364)** Cross-sectional online survey 5-point Likert scale ranging from strongly agree to strongly disagree	I. Familiarity and involvement with CHWs II. As a result of working with CHWs, patients are more likely to: 1. Follow my recommendations 2. Show up for scheduled appointments 3. Maintain regular care 4. Better manage their chronic disease 5. Have good birth outcomes 6. Have more effective communication during office visits 7. Have better access to care III. In my experience, CHWs have contributed to: 1. Reduction in the cost of care for high risk or high-cost patients 2. Reduction in the cost of care for NON-high risk or high-cost patients 3. Improved health outcomes for high risk or high-cost patients 4. Improved health outcomes for NON-high risk or high-cost patients 5. Prevention of high risk or high-cost health conditions IV. In my experience, CHWs have saved me time: 1. Arranging clinical referrals and follow-up for patients 2. Arranging social service referrals for patients 3. Educating patients on disease management 4. Educating patients on health promotion (i.e., nutrition and physical activity) 5. Educating patients on healthy childbirth V. Overall, how do CHWs in your organization work with the primary care team: 1. Meeting regularly with primary care staff 2. Regularly receiving patient referrals or assignments from primary care staff (for needed education sessions or home visits) 3. Providing interpreting services VI. What would make you more likely to utilize CHWs as part of the health care team: 1. More evidence that CHWs improve health outcomes 2. If CHWs services were reimbursed (i.e., By Center for Medicare and Medicaid Services (CMS), AHCCCS, third party payers)

*Authors' description of survey and interview guide domains*.

### Health Plan Leadership Interviews

In 2018, we assessed organizational readiness for system change and actual integration of CHWs, including the recruitment, training and onboarding process among Medicaid-contracted health plans. We collaborated with health policy experts from the Arizona Association of Health Plans (AAHP), an alliance of Arizona Medicaid-contracted health plans that represents the policy interests of these plans, and the Arizona Department Health Services Table 1. Together we engaged leadership of all six Medicaid-contracted health plans through 60–75-min, semi-structured qualitative interviews. Through purposive sampling of health plan leadership teams, which often included the chief medical officer (CMO), chief operating officer (COO) and the chief financial officer (CFO), we explored current and projected utilization, recruitment, training, and financing of the CHW workforce among other topics described in Table 1. Purposive sampling and inclusion of various leadership team members in the single interview was recommended by our health policy expert partners to ensure comprehensive responses to the domains of the interview guide that may or may not have been known by any one individual leader. Questions were piloted with one health plan leadership team, inclusive of a team of CMO, CEO and CFO, and adjusted to strengthen interview flow and timing, reflect key areas of focus and adapt for changes in Arizona health policy affecting CHWs and health plans. Recruitment occurred through the AAHP partner, who explained the project to health plan leadership during regularly scheduled meetings. Leaders were provided the interview guide and asked to identify members of their team with adequate knowledge to answer the questions. Researchers worked directly with designated health plan liaisons to schedule the interviews, which were facilitated by the same primary interviewer trained in qualitative research methods. Interviews were audio recorded and transcribed verbatim. A team of two research staff, inclusive of the primary interviewer and the primary study lead, used a collaborative analysis approach to first, discuss and identify common themes for the major domains of the interview guide and then to develop a code book, later confirmed by study partners (16). The Community Health Worker Core Consensus (C3) Project's 10 CHW core competencies definitions were used to code for CHW core competencies. Using Atlasti eight qualitative research software, the primary interviewer coded the interviews using the agreed upon codebook. Through a process of consensus, the two researchers met face to face over a series of meetings to interpret the findings, address discrepancies in coding and prepare coding memos which were shared with study partners for final interpretation of results (16, 17). Triangulation of the complementary data sources (survey and interviews) occurred in two phases: (1) first, we created a comprehensive description of the characteristics and other emergent themes found in the provider survey results and the health plan leadership interviews; and then we (2) compared and contrasted the relationships and identified commonalities and differences in knowledge, beliefs, attitudes and professional experience with the CHW workforce, the perceived influence of CHWs on patient outcomes and the quality and cost of care, and the mechanisms for CHW integration within the health care team. These interpretations were again shared back with research partners for interpretation recommendations.

## Results

Results will be presented by selected survey and interview guide topics outlined in Table 1. A total of 364 Arizona licensed providers completed the survey in its entirety. Given our focus on licensed providers with experience with CHWs, our analysis includes only the 245 (70%) providers who reported direct or indirect involvement with a CHW. Among these providers, 91% (*N* = 223) were somewhat to extremely familiar with CHWs. Physicians, Physicians' Assistants and Nurse Practitioners accounted for 65% (*N* = 160) of the sample. Approximately 56% (*N* = 137) of the sample were currently employed in a clinical setting designated as a patient center medical home model (PCMH). Participants represented the breadth of health care contexts: 39% (*N* = 88) of participants were employed in a FQHC, 29% (*N* = 66) in Indian Health Service/638 Clinic/Hospital, and 32% (*N* = 74) in a group, solo practice, managed care, or hospital-based practice. We coupled this survey with interviews with 16 (*N* = 16) individuals representing leadership roles within six AHCCCS Complete Care (ACC) contracted health plans, as of October 1, 2018. Participants held positions of chief management, medical, financial and quality assurance officers. Over half of health plan leaders interviewed were employed with the health plan for at least 5 years. Approximately 90% of health plan leaders interviewed were moderately to extremely familiar with CHWs; those with less familiarity with CHWs were those in financial management roles.

### Integration Within Systems and Teams

Among licensed providers, we assessed ways in which they believed CHWs were integrated into the clinical care team Table 2. Respectively, 48, 68, and 52% of providers surveyed reported CHW integration, taking one or more of the following forms: CHWs receive ongoing referrals or assignments by provider staff (*N* = 166); CHWs have regular meetings with clinical care team staff (*N* = 107); and or CHWs provide translational or language interpretation services for patients (*N* = 126). We assessed CHW integration within health plans by first asking participants to describe the known roles CHWs play within their organization and within their contracted provider networks. Health plan leaders interviewed described nearly all of the 10 CHW core competencies in their descriptions of CHW integration Table 3. Most often, leaders described CHW integration within health plan systems and within care teams as connecting members to community resources, providing health behavior education, assisting in health system navigation, and conducting outreach to hard-to-reach health plan members. Health plan leaders emphasized the importance of CHWs' ability to address social determinants of health by connecting members to community and health resources. In one instance, leaders from Health Plan A described expanding the role of the CHWs from a limited, telephone-based, patient navigation role — to a broader role focused on the social determinants of health within the home and clinic. According to leadership, CHWs are now employed as part of an interdisciplinary care team that works with high-need members. CHWs on this team are highly integrated into the health care team and have a variety of roles including connecting members to community resources to address social determinants of health and provide health behavior support and education. Leaders described in detail:

“*They [CHWs] do a lot of resource finding […] a lot of the social determinants they are focused on, but also chronic disease support and management, goal setting, SMART goal setting with patients around their medication adherence, their disease management, their wellness and making sure they make their appointments, making sure they know how to use the medical system, they might accompany people to a medical or behavioral health appointment and then they support the other roles of the team. They are the support system for the nurse practitioner, the clinical pharmacist, the behavioral health specialist and the nurse…”*

**Table 2:** Forms of CHW integration and licensed health care provider attitudes and beliefs about the impact of CHW integration patient health outcomes, provider time and cost of care.

	**Agree/Strongly agree**				
	**Total**	**FQHC/Clinic**	**Health practice^a^**	**IHS/Tribal clinic**	**Other^b^**
**Forms of CHW integration with clinical care team**					
Meeting regularly with primary care staff	44 (107/245)	53 (49)	56 (35)	21 (14)	43 (9)
Regularly receiving patient referrals or assignments from primary care staff^c^	68 (166/245)	75 (70)	71 (45)	56 (38)	62 (13)
Provide interpreting services	52 (126/243)	50 (46)	60 (37)	46 (31)	57 (12)
**As a result of working with a CHW, CHWs have contributed to:**					
Good birth outcomes	52 (123/237)	50 (43)	55 (33)	49 (34)	59 (13)
Prevention of high risk or high-cost health conditions	65 (160/247)	73 (68)	65 (41)	54 (37)	64 (14)
Improved health outcomes for high risk or high-cost patients	69 (170/247)	75 (70)	67 (42)	64 (44)	64 (14)
Improved health outcomes for NON-high risk or high-cost patients^c^	61 (151/247)	73 (68)	54 (34)	56 (39)	45 (10)
Reduction in the cost of care of high risk or high-cost patients	55 (135/247)	60 (56)	59 (37)	43 (30)	55 (12)
Reduction in the cost of care of NON-high risk or high-cost patientsz^c^	47 (115/247)	57 (53)	46 (29)	33 (23)	45 (10)
**CHWs have saved provider time in:**					
Arranging clinical referrals and follow-up for patients	65 (161/247)	69 (64)	73 (46)	55 (38)	59 (13)
Arranging social service referrals for patients ^c^	69 (171/247)	71 (66)	81 (51)	52 (36)	82 (18)
Educating patients on disease management	70 (174/247)	70 (65)	67 (42)	74 (51)	73 (16)
Educating patients on health promotion	77 (190/247)	80 (74)	70 (44)	80 (55)	77 (17)
Educating patients on healthy childbirth ^c^	52 (122/236)	55 (48)	53 (32)	42 (28)	64 (14)
**As a result of working with a CHW, patients are more likely to:**					
Follow my recommendations	76 (186/246)	78 (72)	76 (48)	71 (49)	77 (17)
Show up for scheduled appointments	74 (184/247)	73 (68)	75 (47)	74 (51)	82 (18)
Maintain regular care	76 (187/247)	80 (74)	76 (48)	70 (48)	77 (17)
Better manage their chronic disease	72 (178/246)	78 (72)	68 (43)	68 (47)	73 (16)
Have more effective communication during office visits	64 (158/246)	73 (68)	61 (38)	55 (38)	64 (14)
Have better access to care	80 (196/246)	85 (79)	73 (45)	77 (53)	86 (19)

a *Health Practice include solo practice, group practice, managed care organization and behavioral health*.

b *Other includes items were sites that did not fit into any other pre-defined category*.

c *Statistically significant at the 0.05 level*.

**Table 3:** Community health worker core competencies and roles utilized within arizona medicaid-contracted health plans.

**Roles^a^**	**Health**	**Health**	**Health**	**Health**	**Health**	**Health**
	**Plan A**	**Plan B**	**Plan C**	**Plan D**	**Plan E^b^**	**Plan F**
Cultural Mediation among individuals, Communities, and Health and Social Service Systems	✓	✓	✓	✓		✓
Providing Culturally Appropriate Health Education and Information	✓	✓	✓	✓		✓
Care Coordination, Case Management, and System Navigation	✓	✓	✓			✓
Providing Coaching and Social Support	✓		✓	✓		✓
Advocating for Individuals and Communities	✓	✓				✓
Building Individual and Community Capacity	✓	✓		✓		✓
Providing Direct Service	✓	✓	✓	✓		✓
Implementing Individual and Community Assessments						✓
Conducting Outreach	✓	✓	✓	✓		✓
Participating in Evaluation and Research						

*Community Health Worker Core Consensus (C3) Project: 2016. Recommendations on CHW Roles, Skills, and Qualities. Access at http://bit.ly/2wzz2oe; Authors' analysis of data from the Integration and Financing of Community Health Worker. Workforce in AHCCCS Health Plans interviews, 2018*.

a *The Community Health Worker Core Consensus (C3) Project: A report on the C3 Project Phase 1 and 2. Together Leaning Toward the Sky; 2019. https://www.c3project.org/*.

b *Health Plan utilizes peer support workers only*.

*A ✓ indicates that the given role (identified as a core CHW role by the C3 Project) is performed by CHWs employed by the health plan*.

Participating leaders from Health Plan B further described how they employ CHWs in a variety of roles including patient navigation, patient advocacy, health education, as members of the interdisciplinary health care team, and in conducting outreach. Much of their work involves in-person interactions with members, connecting them with community and health resources to address social barriers to health. In Health Plan F, CHWs were described to act as integrated members of their care management and member engagement teams and support addressing social determinants of health including transportation, housing, food insecurity, and employment. It is notable that only one health plan leader described the CHW core competency of implementing individual and community assessments. This core competency could help plans identify and address population level determinants of health.

### CHW Identification, Recruitment and Training

Health plan leaders described in detail the ways in which they identify and recruit CHW team members. Recruitment was generally described as occurring through referrals from current employees and from partner organizations or training programs. The most commonly required qualifications included a health-related degree or certification, computer skills, communication skills, experience in a health care setting, and familiarity with community resources. Health Plan A leadership identified a variety of skills they look for in a CHW, including customer care experience, computer skills, basic familiarity with medical records, communication skills, and being both bilingual and bicultural. In terms of recruitment, the plan specifically mentioned FQHCs that often identify outstanding existing employees and provide them with the training to become CHWs. Health Plan F leaders also identified several key skill areas they look for in CHWs, including a degree or certification related to health or social services (e.g., CNA, or BA in social work), some experience in the health care setting, familiarity with the culture and resources of the community served, and knowledge of or desire to learn about both motivational interviewing and trauma informed approaches. The recruitment process for CHWs started with internal job postings, as well as external sites connected to the health plan, and taking referrals from current CHWs. Health Plan B sought CHW applicants who had lived experience in the areas of health care navigation, care giving, or community services. The plan preferred to hire CHWs with CHW certification (12), and emphasized their willingness to support an employee to become certified. Recruitment primarily happened through community partners, word of mouth, and certification programs that used their plan as a practicum site.

Leaders at Health Plan E, which at the time did not employ CHWs, described the basic qualifications for Peer Support Specialists (PSS), a type of CHW workforce currently recognized by the Arizona Department of Behavioral Health Services (DBHS) and whose services are reimbursable by Arizona Medicaid. They described the qualifications for PSS as having “lived experience,” meaning personal experience – as opposed to formal training – with the criminal justice system, with alcohol or substance use and or history of mental illness; being over the age of 21; and having a fingerprint clearance card. Similarly, Health Plan C leaders spoke specifically about PSS, and required these same qualifications, in addition to good computer and communication skills. They identified potential PSS mainly through current PSS or case managers. FQHCs in Health Plan C provide network identifies CHWs by their active role in the community. The health plan also described their recent contractual relationship with a third-party agency that hires, trains, and pays for CHWs serving members in one of their service areas. Health Plan D, which also does not currently utilize CHWs but planned to do so in the future, stated that they would look to a community partner that specializes in CHWs, such as the Arizona CHW professional association, to take the lead on determining the specific qualifications for CHWs. In addition, they would prefer for all CHWs to have certification, for liability reasons.

Training requirements and opportunities for CHW integration varied widely among health plans. Several plans preferred to hire CHWs with formal certification, however it was not required at the time of the interview. While one plan provided extensive internal training for CHWs, others described a more basic training (or retraining for current employees) on health plan systems and community resources. Health Plan F required extensive internal training for CHWs, using evidence-based curricula developed by their national team. CHWs took part in a 2-week training specifically on the roles of a CHW, followed by a 3-week preceptor ship. Each month the CHWs participated in grand rounds with the health plan's national medical director and received training on a specific topic such as depression. CHWs received extensive workforce safety training and trainings related to disease management, health behavior, and community resources. The health plan also used “field-based ride-alongs” to train new CHWs through direct observation of experienced CHWs – an opportunity also available to health plan leaders and management. Health Plan A leaders described that within some of their contracted FQHCs existing employees are often recruited and re-trained to become CHWs. At the time, Health Plan E only employed PSS, who were required to go through a state approved Peer Support Employment Training Program, one of which was offered internally at the health plan (Since 2012, the state has required that all PSS pass the state-approved training in order to have their services billed through Medicaid). Once credentialed, PSS went through basic employee trainings in areas such as HIPAA. Leaders from Health Plan C, which only employed PSS at the time of the interview, discussed their efforts around creating future CHW positions that would require CHW certification as well as training on electronic health record systems. They described working with several Arizona community colleges to develop and improve their curriculum for CHW certification to include behavioral and mental health components. In addition, the health plan had financed the training of more than 50 people to attend the CHW programs at these community colleges.

### CHW Integration Contributions to Quality of Care

We assessed how CHW integration contributed to the quality and cost of care among providers and health plan leaders Table 2. Among licensed health care providers surveyed, ~87% (*N* = 214) believed CHWs have a positive impact on patient care. In terms of quality and continuity of care, respondents reported that patients who have CHW contact were more likely to follow their recommendations (76%, *N* = 186), show up for scheduled appointments (74%, *N* = 184), maintain regular care (76%, *N* = 187), and better self-manage chronic disease (72%, *N* = 178). Respondents perceived CHWs to increase patient access to care (80%, *N* = 197) and enhance the efficacy of patient-provider communication (64%, *N* = 158) Table 2. Providers surveyed believed CHWs saved them time specifically through arranging clinical (65%, *N* = 161) and social referrals for patients (69%, *N* = 171), as well as educating patients on disease management (70%, *N* = 174) and health promotion (77%, *N* = 190). These attitudes and beliefs were consistent across all health care contexts.

Among health plan leaders, CHW integration was believed to impact the quality of care in two main areas: medical and social. In the first area, several health plan leaders described the positive impact CHWs have on member outreach and engagement, as well as on the utilization of preventative and primary care. The reduction of emergency services was also cited as a major benefit of CHW involvement. CHW impact on housing and justice involvement was also described. Health plan leaders emphasized that the value of CHWs was difficult to measure by standard metrics and that their value was in part due to their unique understanding of the community served.

Health Plan a noted two main areas of value in terms of CHW integration: “member outreach” and the completion of “certain preventative services.” However, they emphasized that the positive impact that CHWs have on the quality of patient care often did not align within the “metrics that CMS or other large agencies have come up with.” Health Plan B had found that CHWs had a significant impact on improving quality of care for members, particularly in the area of preventative services. They measured CHW integration impact on quality of care by focusing on what happened when certain social barriers (e.g., unemployment, lack of transportation, housing insecurity) were removed as a result of a CHWs efforts. Health Plan C leaders explained how CHW integration helped to “normalize” the utilization of health care among populations that traditionally are reluctant to seek medical services. Health Plan D described observing positive impacts in lowering emergency department admissions, reducing involvement in the justice system, and increasing housing for homeless plan members. Health Plan E, D and F leaders believed CHW lived experience made them extremely effective advocates for their clients within the health system and the community.

### CHW Integration Contributions to Cost of Care

Approximately two-thirds of licensed health care providers surveyed believed that CHW integration contributed to the prevention of high risk or high-cost health conditions (65%, *N* = 160), improved health outcomes for high risk and high-cost patients (69%, *N* = 170) and saved them time with their patients (66% across all provider categories). Table 2. Health plan leaders concurred with this belief and described a significant reduction in member costs due to increased utilization of primary and preventative services and reduced utilization of emergency and inpatient services. In addition, CHWs were believed to provide high value, low-cost services as part of the health care team.

“*As the price goes up then the value equation gets a little more challenged, because in some ways you're just trying to replace a higher cost, you don't need an RN or an LPN or someone with a given license to do the work, it might be done more effectively by a CHW, but part of that value equation is that they are a lower cost staff member.”*

Health Plan B found cost savings through the use of CHWs in large part because CHW activities tend to reduce utilization of expensive and sometimes unnecessary services such as emergency room visits, hospital inpatient admissions, and rapid readmissions. Leaders from Health Plan D had not formally evaluated cost savings of CHWs, largely because their reasoning behind CHW integration was focused on improving quality of care rather than cost. With that said, Health Plan C, D and E had observed a general reduction in cost around emergency department utilization, treatment adherence and hospital inpatient admissions, as members working with CHWs were more likely to seek primary care and preventative services. For Health Plan C, members involved with Peer Support Services were shown to have fewer inpatient events, less justice involvement, and reduced use of emergency services. Health Plan E leaders described these impacts as a result of CHWs preemptively reaching out to high needs members and connecting them with preventative health care services and addressing social barriers before a crisis occurs.

### Financing CHW Integration

Finally, we assessed how health plans financed CHW integration within systems and teams. Health plans described four models used to finance CHW integration; administrative or operations budget/dollars, grant funding, value-based payment arrangements and Arizona Medicaid billing codes (PSS only). Health plan leaders indicated that their plan and provider networks often used more than one of these finance models depending on the CHW's role and position in the health care team. Several plans utilized administrative funds to directly employ CHWs, noting however that this was not a particularly sustainable model and would not be cost-effective for providers. Administrative funds were used occasionally to pilot programs, with the goal of ultimately moving toward value-based purchasing. In Health Plans B and F, CHWs were employees paid through operations or administrative budgets, while at the provider network level, CHWs were paid through value-based contracts. Leaders at Health Plans D and C described providers in their networks had funded CHW positions through grant funding, which one leader explained was often restrictive and resulted in CHW positions that were short-term and frequently narrow in scope. Many health plan leaders described the utility of value-based purchasing to allow contracted providers to achieve high quality outcomes through creative means, such as hiring non-clinical team members like CHWs. One plan leader explained their perspective on value-based purchasing, which is focused on achieving health outcomes rather than services provided:

“*I think value-based arrangements allow for the use of CHWs because we're just giving a chunk of money and we don't dictate how you use it as long as you're achieving good outcomes. As opposed to the current system where you have to be a professional that can bill for a given unit of service, which is this fee service system. […] the reality of value based is about achieving value at high quality outcomes so it's not dictating the process by which you do that.”*

Health plans employing PSS are able to fund those positions through Medicaid billing codes for services, in addition to some administrative or grant funding for specific community-based projects. Several plans cited the lack of a similar dedicated Arizona Medicaid billing code for CHWs as a challenge to creating sustainable CHW integration systems that utilize the full CHW scope of practice. One leader explained the benefits of a CHW billing code this way:

“*…if they (Arizona Medicaid and the Legislature) got CMS's approval to have a specific code that could only be billed by community health workers then yeah, you would see a flood of CHWs across the state. …but again, if that doesn't happen then really the only recourse is to come along side and shore it up as a health plan with different focused grants… and that's the thing about those grants, you really have to figure out what are you wanting to accomplish and what a community health worker is… sometimes you lose a little bit of what a community health worker is when you have it run through grants that are very focused on very specific populations… Not to say it's a bad thing but I guess it's my longwinded way of saying I support CMS or AHCCCS getting that code, otherwise you get like CHW lite.”*

All health plan leaders noted that it would be “very beneficial to see CHWs to have their own billing code,” to support development of future positions.

### Challenges to Integration Within Systems and Team

Among both Licensed health providers surveyed and health plan leaders interviewed, all discussed several challenges around hiring and integrating CHWs into the health care systems and teams Table 4. They highlighted the lack of “consistent understanding” at the plan and provider network level of CHW competencies, roles and training needs, which impacts the training, placement and supervision of potential CHWs. The CMO of one plan described requiring the members of the care team to “review the roles, the functions [of CHWs] and how they would integrate and work together.” Leaders emphasized the lack of recognition by the state Medicaid system and billing codes as the major barrier for uptake and scale of CHWs. Health plan leaders often emphasized the challenges in assessing CHW integration impact through existing standard metrics set forth by CMS (18).

**Table 4:** Health plan leaders attitudes, beliefs, and values regarding CHW health care systems change and integration.

**Theme**	**Thematic summary**	**Direct participant quotes**
CHW integration contributions to quality of care	**Medical needs** -Meaningful outreach and engagement -Increase utilization of primary care -Increase utilization of preventative care -Decrease utilization of emergency services -Improve treatment adherence -Supportive in meeting Healthcare Effectiveness Data and Information Set (HEDIS) **Social determinants of health needs** -Trusting relationship with health plan member -Normalize health care experience -Decrease involvement with justice system -Housing support -Health plan member advocacy -Identify and remove social barriers to care -Save member lives -Encourage behavior change	“*We have all of these health outcomes that we hold our providers to as far as how many of your members end up going to jail, how many of your members end up in the ER regularly, that sort of thing and our providers know they can greatly reduce all of those additional costs with a team of appropriately staffed peer or community health workers.”* *“We have found that for every social barrier that is removed through a community health worker and tracked through the community impact model, we save $450 in reduced emergency room visits, reduced length of stay in a hospital and reduced rapid readmissions. At the same time, not only is there a cost savings but we have found that there is a significant lift in quality scores when those same social barriers are removed. Members are 1½ - 2½ times more likely to schedule and complete their primary care physician visits, they are nearly 7 times more likely to have a better adult BMI score, they remain more compliant with their diabetes treatment and so on. We have each measure documented on what the list is by removing a social barrier, which is one of the key roles that we ask the community health workers to play.”* *“They are saving the lives of the people that they are working with in one way or another. They either help them find a purpose like a job and they feel alive and they want to be alive, reducing suicides, reducing overdoses, letting people know that they are not alone that they too can make it through this, so I would say the most valuable contribution is the lives of our members.”* “*I think the idea that someone that is a little bit more of a lay person, a little bit more of a peer from the community that is field based that actually sees people face to face offers such a tremendous additional opportunity to the rest of the care team and I say the rest I mean the nurses and the physicians which are more office and less field based. You can't account enough for the value that comes from the direct intervention and seeing and meeting with people in their environment and in their home or at their work to try to get a better handle on the needs that they may have and to get a better understanding of what they are going through relative to the healthcare that we are trying to deliver to them.”*
CHW integration contributions to cost of care	-CHWs are of high value at a low cost -Improve adherence to treatment -Reduce use of emergency services -Reduce hospital inpatient admissions -Enhance early identification of high-cost members	“*As the price goes up then the value equation gets a little more challenged, because in some ways you're just trying to replace a higher cost, you don't need an RN or an LPN or someone with a given license to do the work, it might be done more effectively by a CHW, but part of that value equation is that they are a lower cost staff member.”* *“So reaching out to them instead of waiting for them to come to us and so when I think about cost, identifying people that are higher cost and those are the folks that these teams are often times trying to identify and work with earlier rather than later. That's how they help reduce costs […] If they can positively influence and impact those individuals in the ways we talked about earlier we can hopefully reduce that cycling through those very high cost settings which are not the best or the most appropriate places of care.”* *“It [CHW integration] definitely reduces cost for a number of reasons, number one because it improves adherence and people tend to stay in treatment and follow through with their treatment more, so that reduces relapse, that reduces maybe the utilization of the ED [emergency department services].”*
Financing CHW integration via value-based contracts	-Flexible model that allows provider contractors to achieve high quality outcomes through creative means -Payments to providers tied to patient health outcomes, cost of care, and quality of care (not fee-for-service). -Health Plans support (but do not mandate) CHW integration through value-based agreements with provider. -More sustainable than grant funding.	“*Generally speaking most value-based contracting going forward is going to be tied to outcomes rather than processes. … most health plans are moving away from funding specific processes and dictating what those look like and more funding outcomes. But I can see again agencies having better outcomes with CHWs that will be able to take advantage of value-based payment arrangements because of that, because they are making certain outcomes that they are looking for.”* *“We have value-based payment models that are in place with a number of large groups that are total-cost-to-care based, so if they have an impact on any aspect of cost to an individual, they can potentially net benefit from that as part of those value-based payment models. […] sometimes they'll [the provider] say that CHWs are part of their planned approach and if they don't raise that, we usually raise that as a potential best practice that they should be considering. If they do earn shared savings, we don't typically dictate how they can spend those savings but we encourage them to consider the use of some of those savings that they earn and reinvest it back into their program and CHWs are one of those areas we make recommendations that they consider.”* *“I think value-based arrangements allow for the use of CHWs because we're just giving a chunk of money and we don't dictate how you use it as long as you're achieving good outcomes. As opposed to the current system where you have to be a professional that can bill for a given unit of service, which is this fee service system*.
		*[…] the reality of value based is about achieving value at high quality outcomes so it's not dictating the process by which you do that.”* *“So I would not say we're going to have value based payments and you should be hiring community health workers, that's not the point of value based. We're doing value-based payments and this [value-based payments] allows you all to get creative with using whatever you need to get those best outcomes. “*
Challenges in CHW integration	-Lack of understanding among providers of CHW competencies and training needs -Reliable transportation among CHWs -Needing to recraft CHW positions to accommodate non-traditional candidates -Lack of Medicaid billing codes -Lack of understanding CHW competencies and roles among health care team -Locating individuals with the right combination of CHW competency and confidence to integrate into health care team	“*Community health workers aren't currently recognized in the state and so that honestly causes a lot of challenges for us internally to really develop programs.”* *“I wouldn't call them challenges, just things that we process through and we hire individuals that represent the consumers that we provide services to. We have had to recraft the position so that some of those positions are part time and many of the individuals that we hire are non-traditional so we've had to adjust our employment practices to be able to accommodate special needs in a much more highly specialized fashion.”*

*Authors' analysis of data from the Integration and Financing of Community Health Worker Workforce in AHCCCS*.

*Health Plans interviews, 2018*.

## Discussion

Provider surveys and health plan leadership interviews demonstrate that Medicaid-contracted health plans and their provider networks are rapidly incorporating CHWs into the health systems and clinical care teams. Both sets of health care sector actors were knowledgeable of and highly valued CHW expertise and activities as encompassed under the CHW core competencies. Among health plan leaders, all understood and prioritized the cultural, linguistic and lived experience characteristic of the CHW workforce and made efforts to actively recruit, train and integrate CHWs into clinical and community-based teams to benefit health plan members. CHWs were considered to add value to patients care by conducting effective and culturally salient health plan member outreach. For both providers surveyed and health plan leaders interviewed, such culturally informed outreach and education activities conducted in the home, over the phone and in the clinic have resulted in both anecdotal and empirical evidence of improved access to health care, use of prevention screenings, appropriate use of the health care system, including avoidance of emergency room and hospitalization among members. CHW integration was considered essential to increasing access to primary care, self-management activities and behavioral health support for highly vulnerable health plan members. Such perspectives are critical as decisions about CHW integration is increasingly influenced by internal calculations and demonstration projects, even in the absence of rigorously designed peer-reviewed research (19).

Consistent with the literature, health care providers consider CHWs to be valuable members of health teams who play a vital role in addressing medical and social determinants of health among underserved populations. Health plan leaders were motivated to integrate CHWs in part by reforms in health care financing in the United States which are incentivizing the shift toward a value-based reimbursement structure that reward evidence of favorable medical and social outcomes (20). We found health care sector actors to be supportive of the notion that by utilizing their unique position within their community, coupled with core competency and disease specific training, CHWs can play a significant role in improving patient outcomes and reducing system costs of health care (4, 7, 21). Also consistent with the existing evidence, CHWs embedded within the health care team were described to facilitate patient care coordination between social supports, primary care, and collaboration with public health and social service agencies to improve community outreach, wellness education, and chronic disease management (6, 22). Health Plan leaders and providers surveyed perceived CHW interventions to improve several clinical indicators, (23–25) lower risk factors for chronic disease and mental health (26, 27) and increase medication adherence (25, 28). For many leaders, CHW interventions were thought to contribute to a reduction in emergency department visits, (29–33) while CHW integration into the health care team was consistently considered associated with reductions in health care cost (29, 34–37), and for some health plans, either anecdotal or measured, provided a return on investment per dollar invested in CHW interventions (25, 29, 35, 36). Yet, and very consistent with existing literature, inherent challenges exist within the health care system to optimize CHW integration and financing (7).

### Implications for Health Care Policy and Research

In Arizona, the integration of physical and behavioral health services Mandated by the AHCCCS Complete Care (ACC) contract provides a policy window of opportunity to advance and sustain the CHW workforce in contracted health plans (38). Health plan leadership expect that the ACC will fundamentally expand the need for CHWs and their core services, as plans take on an expanded role in meeting membership medical and non-medical needs. ACC contracted health plans with experience in the delivery of behavioral health care through peer supports can provide technical expertise and knowledge transfer related to the process of developing state Medicaid billing codes for non-clinical staff (39, 40). In this same time frame, and partially in response to heightened interest in the documented impacts of the CHW workforce on the U.S. health care system, CHW stakeholders have also converged to more clearly define CHWs' core competencies and roles (41). To adjust provider misunderstanding of CHW roles and competencies and ensure the full range of patient outcomes associated with CHWs, mechanisms to ensure the integrity of the workforce should be cultivated and maintained, including organizational culture and leadership promotive of these values and norms.

The Arizona Legislature recently passed HB2324, which authorized the voluntary certification of community health workers and mandated standardized training of the CHW workforce (12). Arizona Department of Health has established a 9-person advisory council made up of at least 50% CHWs which will be responsible for establishing CHW core competencies, training standards, continuing education requirements and other details related to CHW certification in the state. The Association of Health Plans was strongly in favor of the legislation and the organization's support was pivotal in gaining legislative support in a state that is largely anti-regulation. Arizona health plans expressed the benefit of the HB2324 legislative efforts for voluntary certification, specifically in the opportunity to recruit and retain highly qualified CHW to meet member medical and non-medical needs. Voluntary certification may facilitate reimbursement mechanisms for CHWs, and thus may be an important consideration for financing in other states. In Arizona, certification was the avenue for workforce standardization. Several statewide strategies exist to support the health system's capacity to integrate CHWs into systems of care and clinical care teams, including; (1) Extend Arizona AHCCCS (Medicaid) billing codes to reimburse for CHW services as in the case of Peer Supports; (2) Designate CHWs as a provider and enable CHWs to bill for the full array of CHW core competency services; (3) Monitor CHW innovations emerging from AHCCCS Complete Care (ACC) contracts and CHW voluntary certification legislation; (4) Promote standardized CHW training among health plans and contracted provider networks; (5) Share CHW innovations in training, supervision, hiring, financing and integration within health care teams.

Nationally, Medicaid administrators should leverage existing channels of communication with CMS officials to advance the development metrics that more accurately capture the CHW process and outcomes participants. Health plan leaders in this study identified a clear need for CMS policy change, to be inclusive of metrics related to the social determinants of health, appropriate methodology for measuring CHW integration within systems and teams. The National CHW Common Indicator Project could support with identification of CHW centered process and outcomes measures (42, 43). Without a doubt, the attitudes, beliefs and values of the participants in this research express a resounding call for action on the creation of CHW-specific billing codes across the CMS system. In the paraphrased words of the Institute of Medicine Report, CHWs are effective, and “*If these were the results of a clinical trial for a drug, we would likely see pressure for fast tracking through the FDA; if it was a medical device or a new technology, there would be intense jockeying from a range of start-ups to bring it to market*” (44). This research adds to the mounting evidence of the need for a national strategy toward the development of CHW covered services, billing codes and metrics for CHW integration within systems and teams within the health care sector and beyond.

### Limitations

Our study has several strengths and limitations. The strength of this study lies in the broad based CHW and CHW ally stakeholder engagement to both conceptualize the study and carry out the entirety of the research process. Through our partnerships, we were able to interview the universe of Medicaid health plans in the state. Our limitation includes a non-representative convenience sample of licensed health care providers. Although we engaged in an exhaustive sampling methodology representative of major health care employers of CHWs and several professional associations of licensed providers, our sample was not randomized nor representative of all licensed providers of Arizona. We therefore may be underreporting the experiences of licensed providers with experience with CHWs.

## Conclusions

Our findings demonstrate a high level of organizational readiness and capacity within the Arizona Medicaid system to integrate CHWs in system and teams. Licensed health care providers and health plan leaders demonstrated attitudes, beliefs and values that align with tremendous organizational culture and capacity to transform and innovate the systems and processes for CHW integration and financing. As states move toward standardized CHW certification and the demand for CHWs increases with the transition to coordinated systems of care, health plans and providers will benefit from sharing of best practices, challenges and solutions.

## Data Availability Statement

The raw data supporting the conclusions of this article will be made available by the authors, without undue reservation.

## Ethics Statement

The studies involving human participants were reviewed and approved by University of Arizona Human Subject Review Board. The patients/participants provided their written informed consent to participate in this study.

## Author Contributions

SS co-conceptualized the study and lead the writing of the manuscript. MI, FR, and JG co-conceptualized the study. LO'M and HD facilitated data collection and analysis and interperation of results. NW and HC provided health policy expertise and supported writing and editing. All authors contributed to the article and approved the submitted version.

## Conflict of Interest

The authors declare that the research was conducted in the absence of any commercial or financial relationships that could be construed as a potential conflict of interest.

## References

[1] Sikka R Morath JM Leape L. The quadruple aim: care, health, cost and meaning in work. BMJ Quality Safety. (2015) 24:608. 10.1136/bmjqs-2015-004160 26038586

[2] Bodenheimer T Sinsky C. From triple to quadruple aim: care of the patient requires care of the provider. Ann Family Med. (2014) 12:573–6. 10.1370/afm.1713 25384822 PMC4226781

[3] Balcazar H Rosenthal EL Brownstein JN Rush CH Matos S Hernandez L. Community health workers can be a public health force for change in the United States: three actions for a new paradigm. Am J Public Health. (2011) 101:2199–203. 10.2105/AJPH.2011.300386 22021280 PMC3222447

[4] Brownstein JN Hirsch GR Rosenthal EL Rush CH. Community health workers “101” for primary care providers and other stakeholders in health care systems. J Ambul Care Manage. (2011) 3:210–20. 10.1097/JAC.0b013e31821c645d 21673520

[5] Kangovi S Mitra N Grande D White ML McCollum S Sellman J. Patient-centered community health worker intervention to improve posthospital outcomes: a randomized clinical trial. JAMA Intern Med. (2014) 174:535–43. 10.1001/jamainternmed.2013.14327 24515422

[6] Hartzler AL Tuzzio L Hsu C Wagner EH. Roles and functions of community health workers in primary care. Ann Fam Med. (2018) 16:240–5. 10.1370/afm.2208 29760028 PMC5951253

[7] Rogers EA Manser ST Cleary J Joseph AM Harwood EM Call KT. Integrating community health workers into medical homes. Ann Fam Med. (2018) 16:14–20. 10.1370/afm.2171 29311170 PMC5758315

[8] Katzen A Morgan M. Affordable Care Act Opportunities for Community Health Workers: how Medicaid Preventive Services, Medicaid Health Homes, and State Innovation Models are Including Community Health Workers. (2014). Available online at: www.chlpi.org/wp-content/uploads/2013/12/ACA-Opportunities-for-CHWsFINAL-8-12.pdf (accessed March 5, 2021).

[9] Medicaid CfM. Medicaid and children's health insurance programs: essential health benefits in alternative benefit plans, eligibility notices, fair hearing and appeal processes, and premiums and cost sharing; exchanges: eligibility and enrollment. Washington: Department of Health and Human Services (2013). 23855057

[10] Cox K Jolly S Van Der Staaij S Van Stolk C. Understanding the Drivers of Organisational Capacity. RAND Corporation; Saatchi Institute (2018). Available online at: https://www.rand.org/pubs/research_reports/RR2189.html (accessed April 5, 2021).

[11] AHCCCS Complete Care. The Future of Integrated Healthcare Delivery. (2018). Available online at: https://www.azahcccs.gov/AHCCCS/Initiatives/AHCCCSCompleteCare/ (accessed June 13, 2018).

[12] LegiScanAZ. Arizona HB2324 2018 Fifty-third Legislature 2nd Regular. LegiScan (2018). Available online at: https://legiscan.com/AZ/bill/HB2324/2018

[13] Allen CG Barbero C Shantharam S Moeti R. Is Theory guiding our work? A scoping review on the use of implementation theories, frameworks, and models to bring community health workers into health care settings. J Public Health Manag Pract. (2019) 25:571–80. 10.1097/PHH.0000000000000846 30180116 PMC6395551

[14] Lewis CM Gamboa-Maldonado T Belliard JC Nelson A Montgomery S. Preparing for community health worker integration into clinical care teams through an understanding of patient and community health worker readiness and intent. J Ambul Care Manage. (2019). 42:37–46. 10.1097/JAC.0000000000000261 30499899 PMC6278825

[15] Ajuebor O Cometto G Boniol M Akl EA. Stakeholders' perceptions of policy options to support the integration of community health workers in health systems. Hum Resour Health. (2019) 17:13. 10.1186/s12960-019-0348-6 30777095 PMC6379925

[16] Teufel-Shone NI Williams S. Focus groups in small communities. Prev Chronic Dis. (2010) 7:A67. 20394706 PMC2879999

[17] Patton M. Qualitative Research and Evaluation Methods. 3rd ed St. Thousand Oaks, CA: Sage Publications (2002).

[18] CMS. Quality Measures. (2020). Available online at: https://www.cms.gov/Medicare/Quality-Initiatives-Patient-Assessment-Instruments/QualityMeasures (accessed January 12, 2021).

[19] Rush CH. Return on investment from employment of community health workers. J Ambul Care Manage. (2012) 35:133–7. 10.1097/JAC.0b013e31822c8c26 22415287

[20] Burwell SM. Setting value-based payment goals–HHS efforts to improve U.S. Health Care. N Engl J Med. (2015) 372:897–9. 10.1056/NEJMp1500445 25622024

[21] Rosenthal EL Brownstein JN Rush CH. Community health workers: part of the solution. Health Aff. (2010) 29:1338–42. 10.1377/hlthaff.2010.0081 20606185

[22] Bovbjerg RR Eyster L Ormond BA Anderson T Richardson E. Integrating Community Health Workers into a Reformed Health Care System. Washington, DC: The Urban Institute (2013).

[23] Allen JK Dennison-Himmelfarb CR Szanton SL Bone L Hill MN Levine DM. Community outreach and cardiovascular health (COACH) trial: a randomized, controlled trial of nurse practitioner/community health worker cardiovascular disease risk reduction in urban community health centers. Circ Cardiovasc Qual Outcomes. (2011) 4:595–602. 10.1161/CIRCOUTCOMES.111.961573 21953407 PMC3218795

[24] Culica D Walton JW Harker K Prezio EA. Effectiveness of a community health worker as sole diabetes educator: comparison of CoDE with similar culturally appropriate interventions. J Health Care Poor Underserved. (2008) 19:1076–95. 10.1353/hpu.0.0076 19029738

[25] Margellos-Anast H Gutierrez MA Whitman S. Improving asthma management among African-American children via a community health worker model: findings from a Chicago-based pilot intervention. J Asthma. (2012) 49:380–9. 10.3109/02770903.2012.660295 22348448

[26] Krantz MJ Coronel SM Whitley EM Dale R Yost J Estacio RO. Effectiveness of a community health worker cardiovascular risk reduction program in public health and health care settings. Am J Public Health. (2013) 103:e19–27. 10.2105/AJPH.2012.301068 23153152 PMC3518330

[27] Roman LA Lindsay JK Moore JS Duthie PA Peck C Barton LR. Addressing mental health and stress in medicaid-insured pregnant women using a nurse-community health worker home visiting team. Public Health Nurs. (2007) 24:239–48. 10.1111/j.1525-1446.2007.00630.x 17456125

[28] Roth AM Holmes AM Stump TE. Can lay health workers promote better medical self-management by persons living with HIV? An evaluation of the positive choices program. Patient Educ Couns. (2012) 89:184–90. 10.1016/j.pec.2012.06.010 22770948

[29] Bielaszka-DuVernay C. The 'GRACE' model: in-home assessments lead to better care for dual eligibles. Health Aff. (2011) 30:431–4. 10.1377/hlthaff.2011.0043 21383359

[30] Findley S Rosenthal M Bryant-Stephens T. Community-based care coordination: practical applications for childhood asthma. Health Promot Pract. (2011) 12(6 Suppl 1):52s−62. 10.1177/1524839911404231 22068360

[31] Gary TL Batts-Turner M Yeh HC Hill-Briggs F Bone LR Wang NY. The effects of a nurse case manager and a community health worker team on diabetic control, emergency department visits, and hospitalizations among urban African Americans with type 2 diabetes mellitus: a randomized controlled trial. Arch Intern Med. (2009) 19:1788–94. 10.1001/archinternmed.2009.338 19858437 PMC5675128

[32] Johnson D Saavedra P Sun E Stageman A Grovet D Alfero C. Community health workers and medicaid managed care in New Mexico. J Community Health. (2012) 37:563–71. 10.1007/s10900-011-9484-1 21953498 PMC3343233

[33] Peretz PJ Matiz LA Findley S Lizardo M Evans D McCord M. Community health workers as drivers of a successful community-based disease management initiative. Am J Public Health. (2012) 102:1443–6. 10.2105/AJPH.2011.300585 22515859 PMC3464827

[34] Brown HS Wilson KJ Pagan JA Arcari CM Martinez M Smith K. Cost-effectiveness analysis of a community health worker intervention for low-income hispanic adults with diabetes. Prev Chronic Dis. (2012) 9:E140. 10.5888/pcd9.120074 22916995 PMC3475531

[35] Esperat MC Flores D McMurry L Feng D Song H Billings L. Transformacion para salud: a patient navigation model for chronic disease self-management. Online J Issues Nurs. (2012) 17:2. 22686110

[36] Felix HC Mays GP Stewart MK Cottoms N Olson M. The care span: medicaid savings resulted when community health workers matched those with needs to home and community care. Health Aff. (2011) 30:1366–74. 10.1377/hlthaff.2011.0150 21734212 PMC3182460

[37] Krieger JW Takaro TK Song L Weaver M. The seattle-king county healthy homes project: a randomized, controlled trial of a community health worker intervention to decrease exposure to indoor asthma triggers. Am J Public Health. (2005) 95:652–9. 10.2105/AJPH.2004.042994 15798126 PMC1449237

[38] Kingdon JK. The reality of public policy making. In: Danis M Clancy CM Churchill LR, editors. Ethical Dimensions of Health Policy. New York, NY: Oxford University Press (2005). p. 97–116.

[39] Blash LCK Chapman S. The Peer Provider Workforce in Behavioral Health: A Landscape Analysis - Report-Peer_Provider_Workforce_in_Behavioral_Health-A_Landscape_Analysis.pdf. (2015). Available online at: https://healthworkforce.ucsf.edu/sites/healthworkforce.ucsf.edu/files/Report-Peer_Provider_Workforce_in_Behavioral_Health-A_Landscape_Analysis.pdf (accessed March 5, 2021).

[40] AHCCCS. Peer Recovery Support Training Credentialing Supervision Requirements. 963 Peer and Recovery Support Training, Credentialing and Supervision Requirements. AHCCCS Medical Policy Manual (2021). Available online at: https://www.azahcccs.gov/shared/Downloads/MedicalPolicyManual/900/963.pdf

[41] Rosenthal L Rush C Allen C. Understanding Scope and Competencies: A Contemporary Look at the United States Community Health Worker Field. (2016). Available online at: http://files.ctctcdn.com/a907c850501/1c1289f0-88cc-49c3-a238-66def942c147.pdf?ver=1462294723000 (accessed March 5, 2021).

[42] Kieffer E PG Wang P Garcia L Maes K Allen C Wiggins N. Community Health Worker Common Indicator Summit: Executive Summary Preceding. (2016). Available online at: https://nachw.org/chw_resources/community-health-worker-common-indicator-summit-executive-summary-precedings/ (accessed March 5, 2021).

[43] Kieffer E PG Wang P Rodriguez Garcia L Maes K Allen Wiggins N. National Common Indicators Project Collaborative. Available online at: http://www.michwa.org/common-indicators-project-2/#National (accessed March 5, 2021).

[44] Pittman MSA Broderick A Barnett K. Bringing Community Health Workers into the Mainstream of U.S. Health Care. (2015). Available online at: https://nam.edu/wp-content/uploads/2015/06/chwpaper3.pdf (accessed March 5, 2021). 10.31478/201502c

